# Change Detection in SAR Images Based on the ROF Model Semi-Implicit Denoising Method

**DOI:** 10.3390/s19051179

**Published:** 2019-03-07

**Authors:** Xuemei Lou, Zhenhong Jia, Jie Yang, Nikola Kasabov

**Affiliations:** 1 College of Information Science and Engineering, Xinjiang University, Urumuqi 830046, China; jzhh@xju.edu.cn; 2 Institute of Image Processing and Pattern Recognition, Shanghai Jiao Tong University, Shanghai 200240, China; Jieyang@sjtu.edu.cn; 3 Knowledge Engineering and Discovery Research Institute, Auckland University of Technology, Auckland 1020, New Zealand; nkasabov@aut.ac.nz

**Keywords:** remote sensing image, ROF model semi-implicit denoising, PCA fusion, FLICM, change detection

## Abstract

The explicit solution of the traditional ROF model in image denoising has the disadvantages of unstable results and requiring many iterations. To solve the problem, a new method, ROF model semi-implicit denoising, is proposed in this paper and applied to change detections of synthetic aperture radar (SAR) images. All remote sensing images used in this article have been calibrated by ENVI software. First, the ROF model semi-implicit denoising method is used to denoise the remote sensing images. Second, for the denoised images, difference images are obtained by the logarithmic ratio and mean ratio methods. The final difference image is obtained by principal component analysis fusion (PCA fusion) of the two difference images. Finally, the final difference image is clustered by fuzzy local information C-means clustering (FLICM) to obtain the change regions. The research results show that the proposed method has high detection accuracy and time operation efficiency.

## 1. Introduction

Change detection of remote sensing images quantitatively analyzes image information at different times in the same area, so as to obtain the change information of the coverage area [1]. Synthetic aperture radar (SAR) is an active remote sensing technology that can collect ground information at any time and under any conditions [2,3]. Remote sensing image change detection technology can assist in updating geographic data, assessing disasters, predicting disaster development trends, and monitoring land use. The processing steps of change detection mainly include image preprocessing, generation of the difference image, detection of the change information, and evaluation of the detection result [4]. However, in SAR imaging processing, the coherent interaction between elementary scatterers on the ground and the electromagnetic waves leads to a multiplicative noise, known as speckle, affecting the SAR images. The interference noise will inevitably be introduced into a SAR image and affect change detection in SAR images [5].

In order to obtain more accurate details of the change, an image preprocessing stage is needed to reduce the noise in the SAR image. There are many methods for image space domain denoising, such as mean filtering [6], median filtering [7], Wiener filtering [8], and Lee filtering [9] methods. Furthermore, image transformation domain denoising methods include the Fourier transform [10], wavelet transform [11], and non-subsampled contourlet transform (NSCT) [12] methods. For change detection in SAR images, an image change detection algorithm based on wavelet fusion of ratio images was proposed in [13], which introduced wavelet domain decomposition into remote sensing image change detection. However, the wavelet transform can only be decomposed in three directions: horizontal, vertical, and diagonal. This means that translational invariance and multi-orientations are not considered, which will cause an image offset error, reducing change detection accuracy. NSCT has good directional selectivity and translational invariance and can effectively improve detection accuracy. Unsupervised detection of different SAR images based on an improved NSCT domain image fusion algorithm was proposed in [14]. The efficiency of the algorithm was greatly improved, but in terms of the details, NSCT had problems with retention. There was still a high amount of noise in the detection results, and the detection accuracy is low.

In recent years, emerging mathematical methods for denoising have received more attention from many scholars. The total variation (TV) model, namely the ROF model, was proposed in [15], and it can effectively reduce noise and preserve the characteristics of the details. An image denoising method based on the ROF model, using the convergence of a central difference discretization, was proposed in [16]. A semi-implicit image denoising algorithm based on the matrix format of the ROF model was proposed in [17]. An image denoising method based on the ROF model using a wavelet transform was proposed in [18]. An improved TV-ROF denoising model based on split Bregman iteration was proposed in [19]. A SAR image change detection method based on an adaptive total variation image denoising algorithm was proposed in [20]. However, the explicit solution of the traditional ROF model in the image denoising process has the disadvantages of unstable solution results and requiring many iterations [21]. To improve the solution of the traditional ROF model, a semi-implicit method is proposed in this paper and applied to the change detection of SAR images.

Based on the above analysis, in this paper a novel change detection algorithm is proposed to improve the image change detection accuracy. In order to obtain more information about the changed region, after semi-implicit denoising with the ROF model for SAR images, this paper combines the advantages of the log ratio method and the mean ratio method to obtain the difference images comprising the change information. Furthermore, this paper uses PCA (principal component analysis) fusion to preserve the characteristics of the significant information in the image and to obtain the final difference image. The fuzzy local information C-means clustering algorithm (FLICM) clusters the final difference image to obtain the changed regions.

## 2. The Proposed Algorithm

Consider two co-registered intensity images T1 and T2, acquired of the same scene at different times, whose sizes are M×N pixels. In order to determine the changed area of the scene over the elapsed time, a novel change detection method is proposed in this paper. The algorithm steps are as follows:

1. use the ROF model semi-implicit denoising method to denoise SAR images T1 and T2;
2. following denoising, obtain the difference images by the logarithmic ratio and mean ratio methods;
3. using the PCA method, fuse the log ratio and mean ratio difference images to obtain the final difference image; and
4. cluster the final difference image by fuzzy local information C-means clustering (FLICM) in order to obtain the change regions.

**Figure 1:**
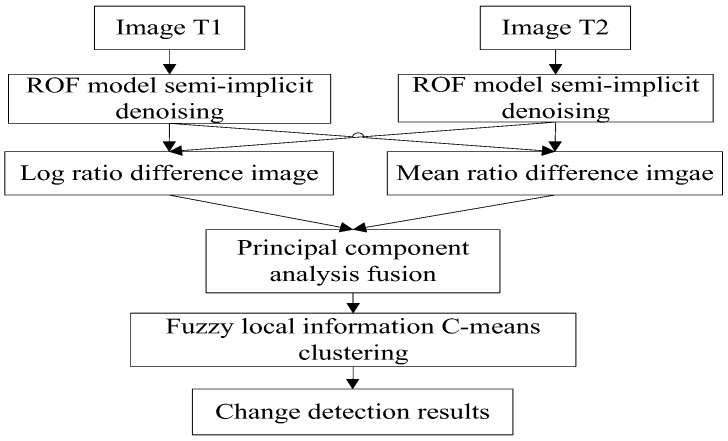
Flowchart of the proposed algorithm for change detection.

The flow chart of the proposed algorithm for change detection is shown in Figure 1.

## 3. Algorithm Introduction

### 3.1. ROF Model Semi-Implicit Denoising

The traditional display ROF model has disadvantages; the results are unstable and require a large number of iterations [22]. Therefore, a semi-implicit discrete iterative solution method is proposed in this paper, with the following steps:

1. establishment of the ROF model; and
2. numerical discretization of the ROF model.

#### 3.1.1. Establishment of the ROF Model

Using the partial differential equation (PDE) for image denoising, a continuous function of slices can be used to approximate the real signal in the image with the edge of the image as the boundary, and the noise in the image is suppressed. Since the approximation is performed over the entire region of the image, it will not cause blurring of the edges of the image. The ROF model is a partial differential equation model based on the total variational method. Total variational image denoising constructs an energy function for the image. Afterward, using the principle that the energy function of the noisy image is larger than that of the original image, the denoised image is obtained by minimizing the energy function.

The traditional variational method uses modern numerical algebra to solve linear equations by introducing least squares fitting, but the effect is not satisfactory. Rudin, Osher, and Fatemi propose a new nonlinear total variation method [15], shown in Equation (1). Then, by solving for the extremum of the energy function, Equation (2), with the variational method, the corresponding Euler-Lagrange equation is obtained, as shown in Equation (3):

(1) $$minimize\int_{\Omega }\sqrt{u_{x}^{2}+u_{y}^{2}}dxdy$$

(2) $$E\left(g\right)=\frac{\lambda }{2}{\iint_{\Omega }\left(f-u\right)}^{2}dxdy+\iint_{\Omega }\rho \left(\sqrt{u_{x}^{2}+u_{y}^{2}}\right)dxdy$$

(3) $$0=\frac{\partial }{\partial x}(\frac{u_{x}}{\sqrt{u_{x}^{2}+u_{y}^{2}}})+\frac{\partial }{\partial y}(\frac{u_{y}}{\sqrt{u_{x}^{2}+u_{y}^{2}}})-\lambda_{1}-\lambda_{2}(f-u),in\Omega$$

where λ is the scale parameter which controls the similarities between the denoised and the original images, Ω is the image area, u represents a noisy image, f indicates the original image without noise, ρ is called the regularized parameter function, which is an incremental function of the gradient and satisfies ρ≥0. In Equation (2), $\frac{\lambda }{2}{\iint_{\Omega }\left(f-u\right)}^{2}dxdy$ is used to restrict the approximation between the noisy image and the original image, while $\iint_{\Omega }\rho \left(\sqrt{u_{x}^{2}+u_{y}^{2}}\right)dxdy$ is used to restrict the smoothness of the image.

PDE denoising uses a piecewise continuous function to approximate the real signal and suppress the noise in the image. PDE denoising is a process of image evolution over time. The time parameter t is introduced as the evolution parameter to solve the parabolic equation. That is, the gradient descent method is used to solve the following evolution equation. The time parameter t is introduced to conveniently represent the denoising process of a noisy image, which can convert Equation (3) into Equation (4). The constraint conditions that satisfy the ROF model are given by Equations (5) and (6):

(4) $$u_{t}=\frac{\partial }{\partial x}(\frac{u_{x}}{\sqrt{u_{x}^{2}+u_{y}^{2}}})+\frac{\partial }{\partial y}(\frac{u_{y}}{\sqrt{u_{x}^{2}+u_{y}^{2}}})-\lambda (f-u),t>0$$

(5) u(x,y,0)=f(x,y)

(6) $$\frac{\partial u}{\partial n}=0,on\Omega$$

When the constraints in Equations (5) and (6) are satisfied, the parameter $\lambda_{t}$ can be obtained from Equations (4) as shown in

(7) $$\lambda_{t}=-\frac{1}{2\sigma^{2}}\left[\iint \sqrt{u_{x}^{2}+u_{y}^{2}}-\left(\frac{f_{x}u_{x}}{\sqrt{u_{x}^{2}+u_{y}^{2}}}+\frac{f_{y}u_{y}}{\sqrt{u_{x}^{2}+u_{y}^{2}}}\right)\right]dxdy$$

#### 3.1.2. Numerical Discretization of the ROF Model

The purpose of the numerical discretization of the ROF model is to optimize the solution of the equation. In time, we adopt a preceding difference scheme, and in space, we use the semi-implicit solution method. The semi-implicit solution method introduces the method of additive operator splitting to numerically discretize the ROF model [23]. The two-dimensional image is computed in the direction of two coordinate axes, and the problem is then transformed into a sum of two one-dimensional problems. The convergence speed is increased by parallel computing.

For images T1 and T2, whose size are M×N pixels, where i=0,1,…,N, j=0,1,…,M, let the positions be $x_{i}=ih$, $y_{i}=jh$, Nh=1. Furthermore, the partial derivatives in the spatial directions are defined as $\Delta_{\pm }^{x}=\pm (u_{i\pm 1,j}-u_{i,j})$ and $\Delta_{\pm }^{y}=\pm (u_{i,j\pm 1}-u_{i,j})$. Let $u^{n+1}=u(x_{i+1},y_{j+1},t_{n+1})$ be the n+1th iteration result and $u^{n}=u(x_{i},y_{j},t_{n})$ be the nth iteration result, where $t_{n}=n\Delta t$. The image boundary conditions are set to $u_{i0j}^{n}=u_{iN}^{n}=u_{i,M-1}^{n}=u_{i,N-1}^{n}$, $u_{Mj}^{n}=u_{M-1j}^{n}$, $u_{Nj}^{n}=u_{N-1j}^{n}$, and $u_{ij}^{0}=u_{0}(x_{i},y_{j})+\sigma \varphi (x_{i},y_{j})$. With these definitions, Equation (4) is numerically approximated by Equation (8):

(8) $$\begin{array}{cc} u_{ij}^{n+1} & =u_{ij}^{n}+\frac{\Delta t}{h}[\Delta_{-}^{x}\left(\frac{\Delta_{+}^{x}u_{ij}^{n}}{\sqrt{{\left(\Delta_{+}^{x}u_{ij}^{n}\right)}^{2}+{\left(minmod\left(\Delta_{+}^{y}u_{ij}^{n},\Delta_{-}^{y}u_{ij}^{n}\right)\right)}^{2}}}\right)+\Delta_{-}^{y}\left(\frac{\Delta_{+}^{y}u_{ij}^{n}}{\sqrt{{\left(\Delta_{+}^{y}u_{ij}^{n}\right)}^{2}+{\left(minmod\left(\Delta_{+}^{x}u_{ij}^{n},\Delta_{-}^{x}u_{ij}^{n}\right)\right)}^{2}}}\right]\\ & -\Delta t\lambda^{n}\left(u_{ij}^{n}-u_{0}\left(x_{i},y_{j}\right)\right)\\ & =u_{ij}^{n}+\frac{\Delta t}{h}\left[\frac{1}{P_{ij}^{n}}u_{i+1,j}^{n}-\left(\frac{1}{P_{ij}^{n}}+\frac{1}{P_{i-1,j}^{n}}\right)u_{ij}^{n}\right)+\frac{1}{P_{i-1,j}^{n}}u_{i-1,j}^{n}+\frac{1}{Q_{ij}^{n}}u_{i,j+1}^{n}-\left(\frac{1}{Q_{ij}^{n}}+\frac{1}{Q_{i,j-1}^{n}}\right)u_{i,j}^{n})+\frac{1}{Q_{i,j-1}^{n}}u_{i,j-1}^{n}]\\ & -\Delta t\lambda^{n}\left(u_{ij}^{n}-u_{0}\left(x_{i},y_{j}\right)\right)] \end{array}$$

(9) $$\lambda^{n}=-\frac{1}{2\sigma^{2}}[\sum_{i,j}\sqrt{{\left(\Delta_{+}^{x}u_{ij}^{n}\right)}^{2}+{\left(\Delta_{+}^{y}u_{ij}^{n}\right)}^{2}}-\frac{\left(\Delta_{+}^{x}u_{ij}^{0})(\Delta_{+}^{x}u_{ij}^{n}\right)}{\sqrt{{\left(\Delta_{+}^{x}u_{ij}^{n}\right)}^{2}+{\left(\Delta_{+}^{y}u_{ij}^{n}\right)}^{2}}}-\frac{\left(\Delta_{+}^{y}u_{ij}^{0})(\Delta_{+}^{y}u_{ij}^{n}\right)}{\sqrt{{\left(\Delta_{+}^{x}u_{ij}^{n}\right)}^{2}+{\left(\Delta_{+}^{y}u_{ij}^{n}\right)}^{2}}}]$$

(10) $$\mathrm{where}\;\{\begin{array}{c} P_{i,j}^{n}=\sqrt{{\left(\Delta_{+}^{x}u_{ij}^{n}\right)}^{2}+{\left(minmod(\Delta_{+}^{y}u_{ij}^{n},\Delta_{-}^{y}u_{ij}^{n})\right)}^{2}}\\ P_{i-1,j}^{n}=\sqrt{{\left(\Delta_{+}^{x}u_{ij}^{n}\right)}^{2}+{\left(minmod(\Delta_{+}^{y}u_{i-1,j}^{n},\Delta_{-}^{y}u_{i-1,j}^{n})\right)}^{2}}\\ Q_{i,j}^{n}=\sqrt{{\left(\Delta_{+}^{y}u_{ij}^{n}\right)}^{2}+{\left(minmod(\Delta_{+}^{x}u_{ij}^{n},\Delta_{-}^{x}u_{ij}^{n})\right)}^{2}}\\ Q_{i,j-1}^{n}=\sqrt{{\left(\Delta_{+}^{y}u_{i,j-1}^{n}\right)}^{2}+{\left(minmod(\Delta_{+}^{x}u_{i,j-1}^{n},\Delta_{-}^{x}u_{i,j-1}^{n})\right)}^{2}} \end{array}$$

Equation (8) is semi-implicit and is expressed in matrix format as

(11) $$u^{n+1}=\frac{1}{2}{\sum_{l}\left[I-2\frac{\Delta t}{h}H{\left(u^{n}\right)}_{l}\right]}^{-1}u^{n}-\Delta t\lambda^{n}(f-u^{n})$$

where I is the identity matrix, and $H=[a_{ij}]$ is an M×N matrix. $H{\left(u^{n}\right)}_{x}$ is the coefficient matrix obtained by differentiating with respect to x, and $H{\left(u^{n}\right)}_{y}$ is the coefficient matrix obtained by differentiating with respect to y, which have the following components:

(12) $$a_{ij}{\left(u^{n}\right)}_{x}=\{\begin{array}{c} \frac{1}{P_{i,j}^{n}},j=i+1\\ \frac{1}{P_{i-1,j}^{n}},j=i-1\\ -(\frac{1}{P_{i,j}^{n}}+\frac{1}{R_{i-1,j}^{n}}),j=i\\ 0,else \end{array}\;\mathrm{and}\;a_{ij}{\left(u^{n}\right)}_{y}=\{\begin{array}{c} \frac{1}{Q_{i,j}^{n}},j=i+1\\ \frac{1}{Q_{i-1,j}^{n}},j=i-1\\ -(\frac{1}{Q_{i,j}^{n}}+\frac{1}{Q_{i-1,j}^{n}}),j=i\\ 0,else \end{array}$$

It can be seen that $a_{ij}{\left(u^{n}\right)}_{x}$ and $a_{ij}{\left(u^{n}\right)}_{y}$ are diagonally dominant tridiagonal matrices [24], and the solution of Equation (11) can be realized in the *X*-axis and *Y*-axis directions simultaneously with parallel processing, to reduce the time needed for the image denoising processing.

### 3.2. Generation of the Difference Images

The ways to generate a difference image include the difference and ratio methods, which involve subtracting and dividing the corresponding pixels in the two images, respectively [25]. The difference and ratio methods are simple to calculate but sensitive to noise in the images, which reduces the accuracy of the detected changes. The difference image constructed by the logarithmic ratio method can enhance the contrast of the changed region by nonlinear stretching on a logarithmic scale, while the unchanged region of the difference image is smoother, which is beneficial for classification [26]. The mean ratio method can effectively enhance the borders of the changed area and small regions of change in the images, and can also prevent the loss of change information [27]. The difference image constructed by the mean ratio method depends only on the relative change in image intensity. It can truly reflect changes in the images and retain more details.

In addition, a single difference image cannot fully represent the difference information. In order to obtain more information about the change regions, this paper combines the advantages of the log ratio and mean ratio methods to obtain difference images containing the change information. The log ratio and the mean ratio difference images are obtained by using Equations (13) and (14), respectively, for images $A_{1}$ and $B_{1}$:

(13) $$ds_{1}(i,j)=\left|\log_{2}\frac{\left(A_{1}(i,j)+1\right)}{\left(B_{1}(i,j)+1\right)}\right|$$

(14) $$ds_{2}(i,j)=1-\min(\frac{u_{1}(i,j)}{u_{2}(i,j)},\frac{u_{2}(i,j)}{u_{1}(i,j)})$$

where $u_{1}(i,j)$ and $u_{2}(i,j)$ represent the mean value of pixel (i,j) in images $A_{1}$ and $B_{1}$, respectively.

### 3.3. Principal Component Analysis Fusion (PCA Fusion)

Principal component analysis (PCA) was first proposed by Karhunen and Loeve, and is also called the K-L transform in mathematics [28]. It is a multi-dimensional orthogonal linear transformation based on statistical properties. The PCA fusion method is mainly based on K-L transformation of the two difference maps and re-projection onto the original coordinate system, so as to transform the gray-scale features of the original image into new features. Finally, the optimized features are used for change detection. The projected features are irrelevant, which suppresses the noise caused by the internal correlations of the image and compresses the original large amount of information into several feature directions. Quantitatively, components of the changed areas are enhanced, components of the non-changed areas are suppressed, and the separability of the changed and the non-changed areas is increased.

In this paper, PCA can be used to preserve the significant information in the image, by applying it to the logarithmic ratio and mean ratio difference images after denoising, and constructing the covariance matrix of the difference image. The eigenvalues and eigenvectors of the covariance matrix are solved, thereby determining the weight coefficients and the final fused image in the difference graph fusion algorithm.

The steps of PCA fusion are as follows:

1. For N images to be fused, treat each image as a one-dimensional vector $x_{k},k=1,2,\ldots ,N$. Construct a data matrix X from the N images to be fused:
  (15) $$X={\left(x_{1},x_{2},\ldots ,x_{N}\right)}^{T}$$
2. Solve for the covariance matrix Cov of X:
  (16) $$Cov=\left(\begin{array}{ccccc} \sigma_{11} & \ldots & \sigma_{1j} & \ldots & \sigma_{1N}\\ \ldots & \ldots & \ldots & \ldots & \ldots \\ \sigma_{i1} & \ldots & \sigma_{ij} & \ldots & \sigma_{iN}\\ \ldots & \ldots & \ldots & \ldots & \ldots \\ \sigma_{N1} & \ldots & \sigma_{Nj} & \ldots & \sigma_{NN} \end{array}\right)$$
  where $\sigma_{ij}^{2}$ is the variance X, and $\bar{x_{i}}$ is the average of the *i*th vector, that is, the average gray value of the *i*th image.
3. Solve for the eigenvalues $\lambda_{1},\lambda_{2},\ldots ,\lambda_{N}$ of the covariance matrix Cov and the corresponding eigenvectors $u_{1},u_{2},\ldots ,u_{N}$. Here, $\lambda_{1}>\lambda_{2}>\ldots >\lambda_{N}$ and the newly obtained feature vectors $Y={\left(y_{1},y_{2},\ldots ,y_{N}\right)}^{T}$ satisfy $Y=U^{T}X$, where $U={\left(u_{1},u_{2},\ldots ,u_{N}\right)}^{T}$, and $C_{y}=diag\{u_{1},u_{2},\ldots ,u_{N}\}$. At this time, $y_{1},y_{2},\ldots ,y_{N}$ are the 1,2,…,n principal components, and $y_{1}$ has the largest variance, which contains a large amount of important information about the difference graph.
4. Determine the weight coefficient $\omega_{i}$:
  (17) $$\omega_{i}=\frac{\lambda_{i}}{\sum_{i=1}^{N}\lambda_{i}}$$
5. Find the final fusion image F:
  (18) $$F=\sum_{i=1}^{N}\omega_{i}S_{i}$$

### 3.4. Fuzzy Local Information C-Means Clustering (FLICM)

A fuzzy local information C-means clustering algorithm (FLICM) was proposed in [29], and the objective function of the traditional FCM algorithm was modified in [30]. The FLICM uses the optimization criterion function $J_{m}$ to calculate the membership of each sample point to the class center. The fuzzy factor $G_{ki}$ is introduced, and the trade-off between image detail information and image noise is done automatically. The neighborhood pixels in the local window have a very flexible effect on the center pixel. The local spatial information is mainly represented by the spatial Euclidean distance between the neighboring pixels and the central pixel. This property can enable the fuzzy factor $G_{ki}$ to better reflect the damping degree of the neighborhood information. This feature can balance the classification tendency of each pixel in the neighborhood window and enhance the robustness of the FLICM algorithm in the presence of noise. The FLICM clustering algorithm uses the optimization criterion function $J_{m}$ shown in Equation (19), and the fuzzy factor $G_{ki}$ shown in Equation (20):

(19) $$J_{m}=\sum_{i=1}^{N}\sum_{k=1}^{C}\left[\mu_{ki}^{n}{\left\|\chi_{i}-v_{k}\right\|}^{2}+G_{ki}\right]$$

(20) $$G_{ki}=\sum_{j=N_{i}}\frac{1}{d_{ij}+1}{\left(1-\mu_{kj}\right)}^{n}{\left\|\chi_{j}-v_{k}\right\|}^{2}$$

where $\chi_{i}$ is the local window center pixel; $\chi_{j}$ is a neighboring pixel near the center pixel *i* of the local window; $d_{ij}$ is the spatial Euclidean distance of pixel *i* and neighboring pixel *j*; $v_{k}$ is the cluster center of class *k*; $\mu_{kj}$ is the membership of the *j*th pixel $\chi_{j}$ to the *k*th class; $\mu_{ki}$ is a fuzzy membership matrix. $v_{k}$ and $\mu_{ki}$ are as defined in Equations (21) and (22):

(21) $$v_{k}=\frac{\sum_{i=1}^{N}\mu_{ki}^{n}\chi_{i}}{\sum_{i=1}^{N}\mu_{ki}^{n}}$$

(22) $$\mu_{ki}=\frac{1}{\sum_{j=1}^{C}\left(\frac{{\left\|\chi_{i}-v_{k}\right\|}^{2}+G_{ki}}{{\left\|\chi_{i}-v_{k}\right\|}^{2}+G_{ji}})(\frac{1}{n-1}\right)}$$

## 4. Experimental Study

In order to verify the effectiveness and to illustrate the practicality of the proposed method, we selected three sets of SAR images, which are well known and often used for comparison.

### 4.1. Description of the Experimental Data

(1) The Bern Dataset

**Figure 2:**
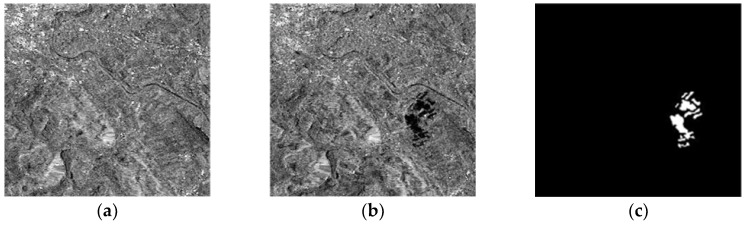
The Bern dataset: (**a**) April 1999; (**b**) May 1999; (**c**) change reference image.

The Bern dataset (Figure 2) is composed of two SAR images from the Bern region of the Swiss capital, acquired by the ERS-2 remote sensing satellite in April 1999 and May 1999. These images have sizes of 301 × 301 pixels, and gray values in the range of 1–256. Figure 2c is a change reference image.

(2) The Coastline Dataset

**Figure 3:**
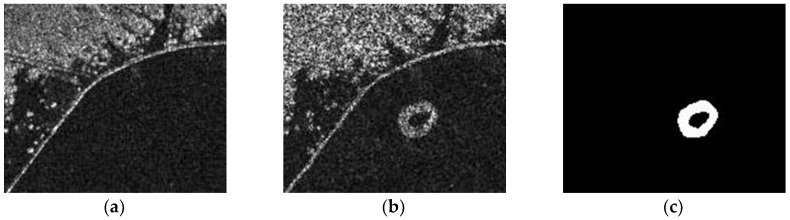
The Coastline dataset: (**a**) June 2008; (**b**) June 2009; (**c**) change reference image.

The Coastline dataset is composed of images of the Yellow River coastline, acquired by the Radarsat-2 remote sensing satellite in June 2008 and June 2009 (Figure 3). These images have sizes of 175 × 147 pixels, and gray values in the range 1–256. Figure 3c is a change reference image.

(3) The Yellow River Dataset

**Figure 4:**
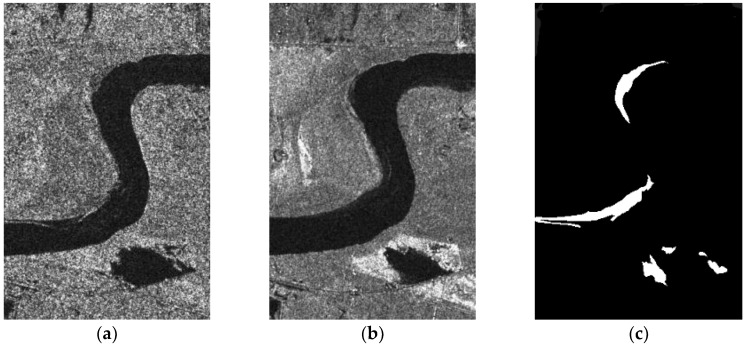
The Yellow River dataset: (**a**) June 2008; (**b**) June 2009; (**c**) change reference image.

The Yellow River dataset is composed of images of the Yellow River estuary, acquired by the Radarsat-2 remote sensing satellite in June 2008 and June 2009 (Figure 4). These images have sizes of 356 × 233 pixels, and gray values in the range 1–256. Figure 4c is a change reference image.

### 4.2. Experimental Parameters

In the semi-implicit denoising process of the ROF model, the parameters of the algorithm design are the scale parameter *λ*, the number of iterations *N*, and the window size *ω*. The scale parameter *λ* is mainly used to control the level of denoising. If the image contains a significant level of noise, *λ* should be a smaller value, while if the image contains a lower level of noise, then *λ* should be a larger value [31]. On the other hand, *N* is a very difficult value to determine. If the number of iterations is set too small, the denoising will be insufficient. If the number of iterations is set too large, not only will the denoising time be increased, but the details will also be lost. At the same time, the size of filtering window directly affects the effect of SAR image filtering. The effect of filtering is poor when the window is too small, and the running time of the algorithm is increased when the window size is too large.

(1) The Scale Parameter *λ*

First, the influence of the scale parameter *λ* will be assessed, based on the percentage of correct classifications (PCC), the kappa coefficient (K), and the change detection time (T) for the change detection results [32]. Other employed objective quantitative indexes include the number of false negatives (FN), the number of false positives (FP), and the overall error (OE), which is the sum of FN and FP. A higher PCC represents a better change detection performance. K is an objective index that is usually used to measure the similarity between the change detection result and the reference image. The ideal value is one, which means the change detection result and the reference image are in complete agreement [33].

**Figure 5:**
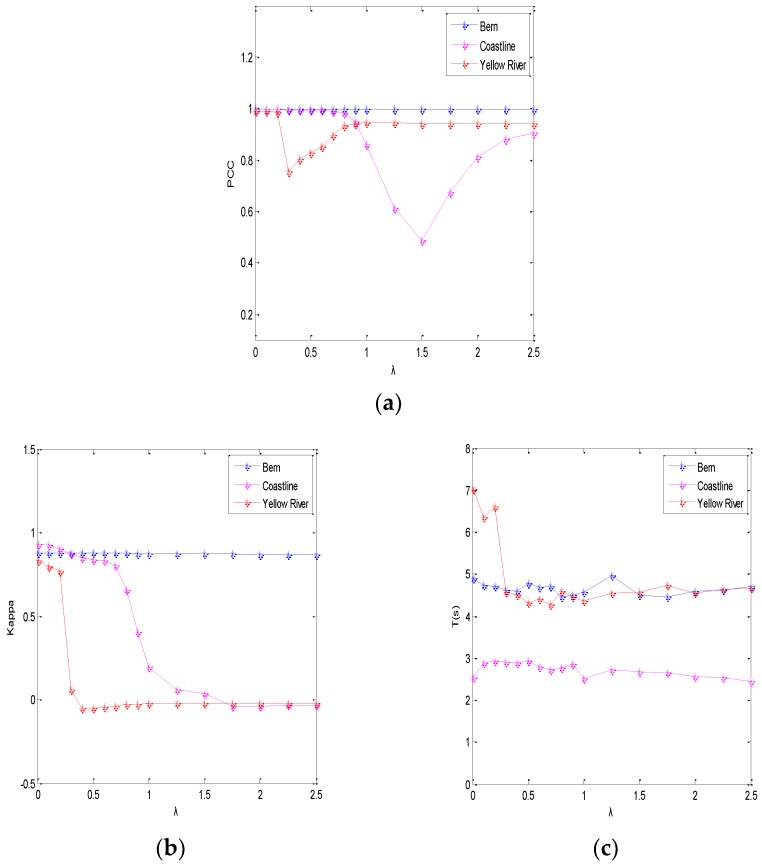
Plots of (**a**) PCC, (**b**) K, and (**c**) T against the scale parameter *λ* on three data sets.

For the Bern SAR images, we fixed the number of iterations to *N* = 2; for the Coastline SAR images, we fixed the number of iterations to *N* = 4; for the Yellow River SAR images, we fixed the number of iterations to *N* = 12. The relationship between the scale parameter *λ* for the three datasets and the objective evaluation indexes are shown in Figure 5. It can be seen that, for the Bern SAR images, *λ* has little effect on the PCC and K; for the Coastline and the Yellow River SAR images, for small values of *λ*, PCC and K are similar. Therefore, in order to balance the values of PCC, K, and T, *λ* is set to 0.4 for the Bern SAR image and to 0.01 for the Coastline and Yellow River SAR images.

(2) The Number of Iterations *N*

**Figure 6:**
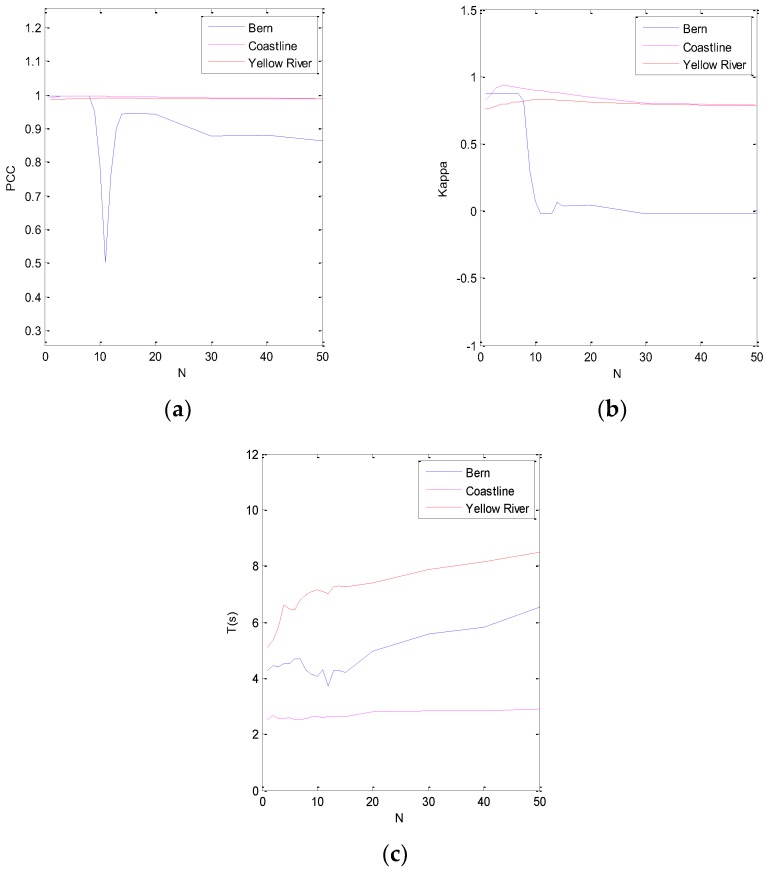
Plots of (**a**) PCC, (**b**) K, and (**c**) T against the number of iterations *N* on three data sets.

The objective evaluation indexes of each dataset as a function of the number of iterations *N* is shown in Figure 6. It can be seen from Figure 6 that, for the Bern SAR images, if 0 < *N* < 8, the corresponding PCC and K are large, while for the Coastline and Yellow River SAR images, the value of *N* has little effect on the objective indexes PCC and K. The influence of *N* on T is shown in Figure 6, and the larger the value of *N*, the longer the processing time. Therefore, in order to balance the values of PCC, K, and T, for the Bern SAR images, we fixed *N* = 2; for the Coastline SAR images, we fixed *N* = 42; for the Yellow River SAR images, we fixed *N* = 12.

(3) The Window Size *ω*

**Figure 7:**
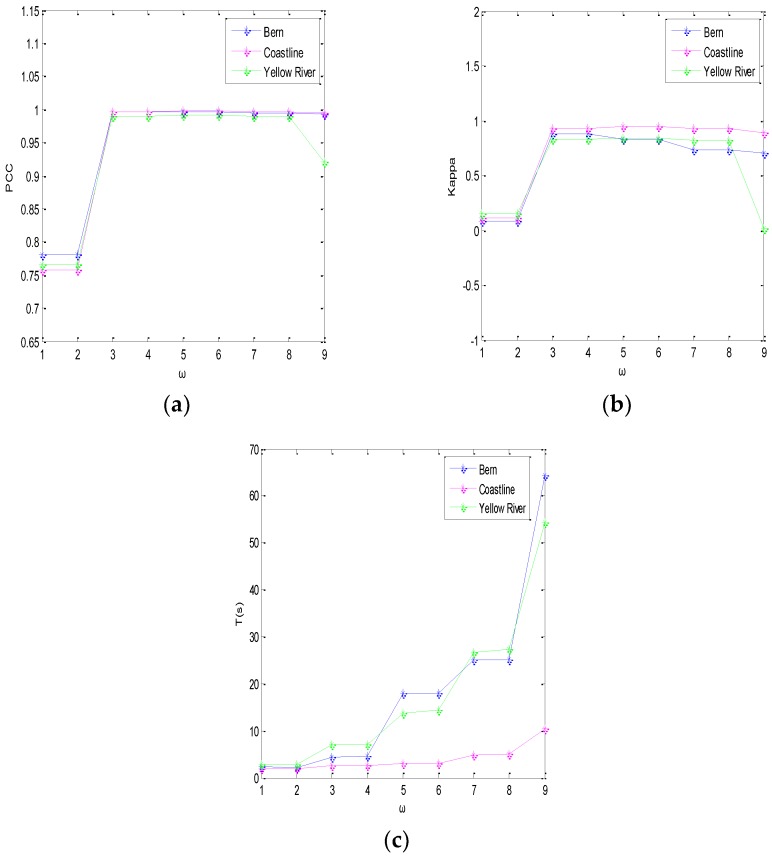
Plots of (**a**) PCC, (**b**) K, and (**c**) T against the window size *ω* on three data sets.

For the fuzzy local information C-means clustering algorithm (FLICM), we next verified the influence of the window size *ω* on the objective indexes PCC, K, and T for the detection results. The relationships between the window size *ω* of each dataset and the objective evaluation indexes are shown in Figure 7. It can be seen that, for the Bern, Coastline, and Yellow River data, the corresponding PCC and K are greatly improved for the window *ω* = 3 × 3, and the detection run time T(s) is reduced. In order to balance the relationship between the size of the window *ω* and PCC, K, and T, the selected window size for the algorithm was fixed at *ω* = 3 × 3.

### 4.3. Experimental Results and Analysis

In order to evaluate the effectiveness of the proposed algorithm, denoted (f) in the following, it was compared with algorithms (a) LEE-FLICM [9], (b) DWT2-FLICM [34], (c) NSCT-FLICM [14], (d) TV-KMEANS [20], and (e) N-FLICM. Among them, we applied the Lee-med filter in algorithm (a) LEE-FLICM instead of the proposed denoising method. On the basis of the proposed algorithm (f), the semi-implicit denoising step of the ROF model was removed in the contrast algorithm (e) N-FLICM. The purpose is to prove that the proposed algorithm (f) can reduce noise and improve the performance of change detection. The paper analyzes both subjective and objective evaluations of the algorithm performance, and its universal applicability.

(1) Subjective Evaluation of Algorithm Performance

**Figure 8:**
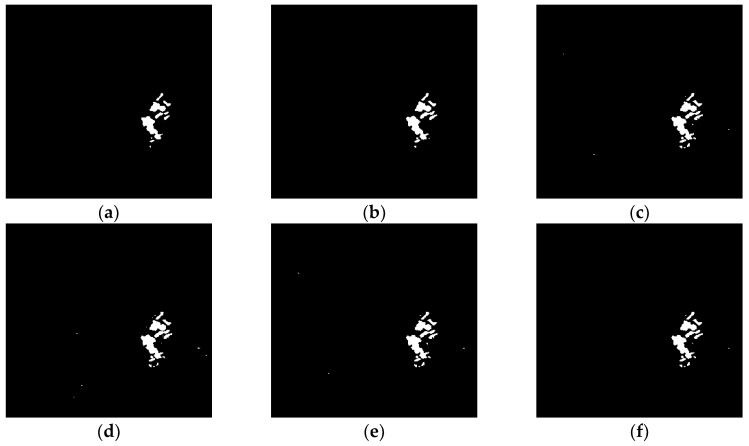
Change detection results of the Bern dataset derived by the (**a**) LEE-FLICM, (**b**) DWT2-FLICM, (**c**) NSCT-FLICM, (**d**) TV-KMEANS, (**e**) N-FLICM, and (**f**) proposed algorithms.

**Figure 9:**
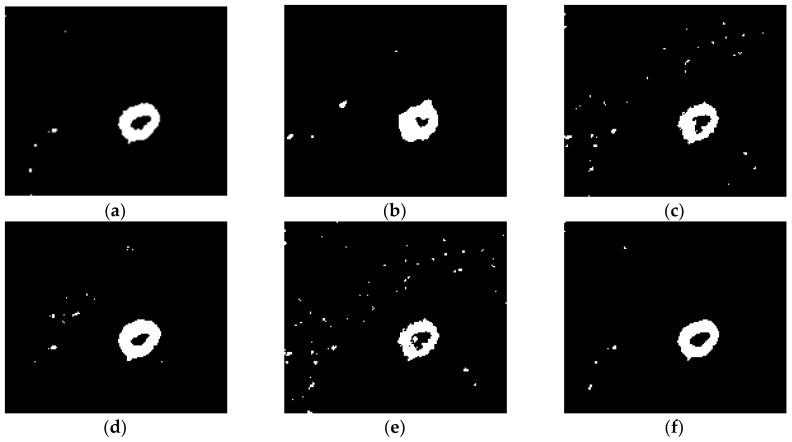
Change detection results of the Coastline dataset derived by the (**a**) LEE-FLICM, (**b**) DWT2-FLICM, (**c**) NSCT-FLICM, (**d**) TV-KMEANS, (**e**) N-FLICM, and (**f**) proposed algorithms.

**Figure 10:**
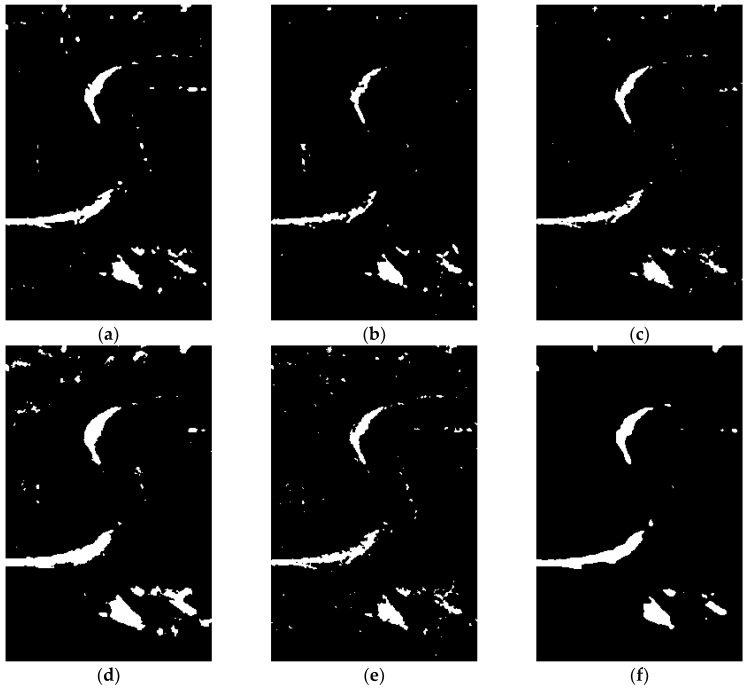
Change detection results of the Yellow River dataset derived by the (**a**) LEE-FLICM, (**b**) DWT2-FLICM, (**c**) NSCT-FLICM, (**d**) TV-KMEANS, (**e**) N-FLICM, and (**f**) proposed algorithms.

The subjective evaluation of the performance of the algorithm is performed by visually analyzing the texture details and noise residuals in the test results shown in Figure 8, Figure 9 and Figure 10. It can be seen from Figure 8, Figure 9 and Figure 10 that, although algorithm (a) LEE-FLICM reduces noise, significant details are lost, and the real change area is not well reflected; algorithm (b) DWT2-FLICM has more missed and false detections, and the change region is not accurately reflected; for algorithms (c) NSCT-FLICM and (d) TV-KMEANS, the change detection image retains the details of the change better, but there is still noise; algorithm (e) N-FLICM contains a high amount of noise and the image details are missing. In all, the proposed algorithm (f) of this paper results in less noise and more details being retained.

(2) Objective Evaluation of Algorithm Performance

In this paper, six objective indicators of change detection are assessed, which are FP, FN, OE, PCC, K and T. The data analysis of the test results is shown in Table 1.

Among the six objective indicators in Table 1, it can be concluded that compared with the algorithm (a) LEE-FLICM, algorithm (b) DWT2-FLICM, algorithm (c) NSCT-FLICM, algorithm (d) TV + Kmeans and algorithm (e) N-FLICM, the algorithm (f) reduces the OE of detection based on the number of FN and FP, which makes the PCC and Kappa coefficient improved; the algorithm not only improves the detection accuracy of the change region in the process of change detection, but also reduces the change detection time, while balancing the detection accuracy and change detection time.

(3) Universal Applicability of the Algorithm

**Figure 11:**
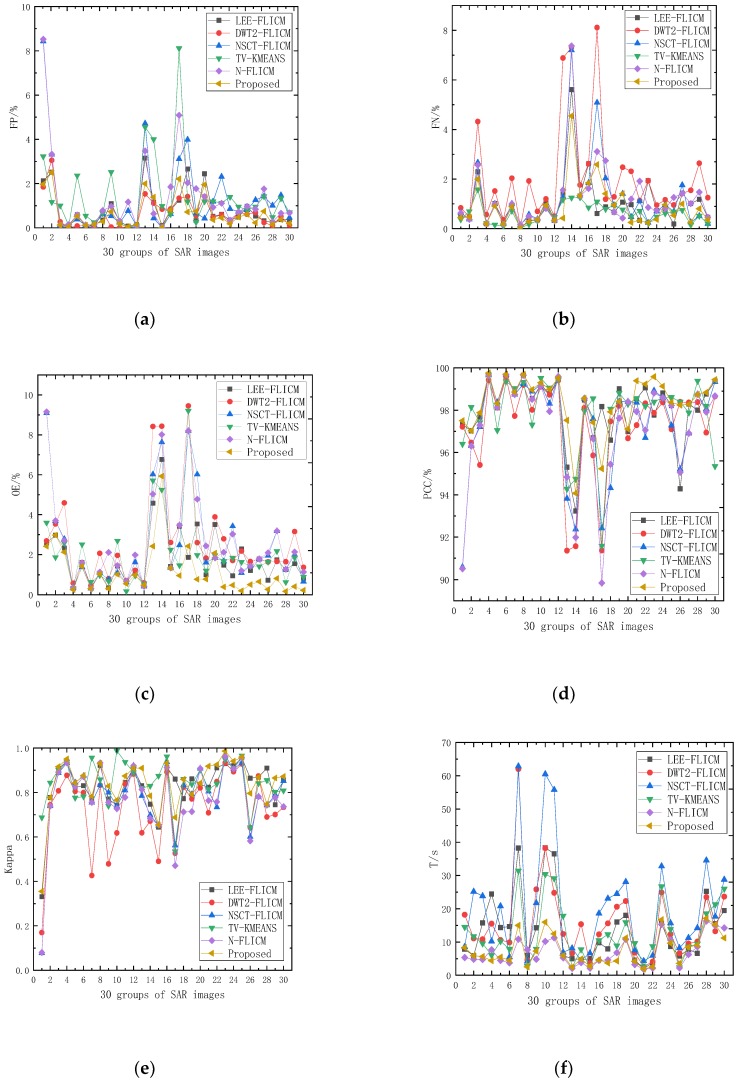
The Objective indicators (**a**) FP/%, (**b**) FN/%, (**c**) OE/%, (**d**) PCC/%, (e) K, and (**f**) T/s for each algorithm on 30 groups of SAR images.

In order to evaluate the general applicability of the method, the above six algorithms were used in data experiments on 30 groups of SAR images, and each group of data was run 20 times to verify the stability of the results of the algorithm. The objective indicators for each algorithm on the 30 groups of SAR images are shown in Figure 11. The average indicators for each algorithm are shown in Table 2.

Among the objective indicators in Figure 11, it can be concluded that, compared with algorithms (a) LEE-FLICM, (b) DWT2-FLICM, (c) NSCT-FLICM, (d) TV-KMEANS, and (e) N-FLICM, algorithm (f) better reduces the false negative rate (FN/%), the false positive rate (FP/%), and the total error rate (OE/%), which further improves the PCC and kappa coefficients. Compared with the previously mentioned algorithms, the fluctuation range of each objective index of the proposed algorithm is smaller, so it has good robustness.

From the analysis of the six average objective indicators in Table 2, compared with the results of the other six algorithms, the proposed algorithm reduces the average total error, OE/%, of detection, which improves the PCC and kappa coefficients. The proposed algorithm (f) achieves a good balance between detection accuracy and algorithm run time, and is more suitable for SAR image change detection.

## 5. Conclusions

In this paper, a new method using semi-implicit denoising of the ROF model is applied to the change detection of remote sensing images. The method can preserve the image texture details and reduce noise. The logarithmic ratio method is combined with complementary information from the mean ratio method in order to obtain a difference image with the change information. PCA is then used to determine a weighted fusion of the two difference images. Finally, the difference image is clustered by fuzzy local information C-means clustering (FLICM) to obtain the change region. The algorithm not only improves the detection accuracy of the changed region, but also balances the change detection accuracy and time. There are many aspects of the proposed methodology that need to be improved. For example, the accuracy of the algorithm can be improved, and the complexity of the algorithm can be reduced. In our future investigations, additional work will be conducted towards these improvements.

## Figures and Tables

**Table 1:** Objective indicators of different detection methods.

Datasets	Method	FP	FN	OE	PCC/%	Kappa	T/s
Bern	LEE-FLICM	34	307	341	99.62	0.8307	14.63
	DWT2-FLICM	107	194	301	99.67	0.8620	9.66
	NSCT-FLICM	106	173	279	99.69	0.8740	5.49
	TV-KMEANS	130	149	279	99.69	0.8767	2.99
	N-FLICM	120	168	288	99.68	0.8710	3.74
	Proposed	100	172	272	99.70	0.8769	4.42
Coastline	LEE-FLICM	88	2	90	99.65	0.9222	5.92
	DWT2-FLICM	189	18	207	99.19	0.8328	4.19
	NSCT-FLICM	159	38	197	99.23	0.8345	4.17
	TV-KMEANS	169	5	174	99.32	0.8582	2.43
	N-FLICM	201	42	243	99.05	0.8019	2.12
	Proposed	74	5	79	99.69	0.9307	2.54
Yellow River	LEE-FLICM	905	284	1189	98.54	0.7703	14.27
	DWT2-FLICM	173	826	999	98.78	0.7489	7.87
	NSCT-FLICM	439	473	912	98.88	0.8001	8.53
	TV-KMEANS	2087	2232	4319	95.88	0.7399	8.23
	N-FLICM	805	409	1214	98.51	0.7555	4.81
	Proposed	608	237	845	98.97	0.8290	7.01

**Table 2:** Average objective indicators of different detection methods.

Method	$\bar{\mathrm{FP}}/\%$	$\bar{\mathrm{FN}}/\%$	$\bar{\mathrm{OE}}/\%$	$\bar{\mathrm{PCC}}/\%$	$\bar{\mathrm{Kappa}}$	$\bar{T}/s$
LEE-FLICM	0.80	1.08	1.84	98.02	0.7860	15.78
DWT2-FLICM	0.65	2.04	2.70	97.29	0.6707	17.96
NSCT-FLICM	1.33	1.22	2.56	97.36	0.7567	21.67
TV-KMEANS	1.45	0.69	3.07	96.63	0.8547	12.78
N-FLICM	1.36	1.27	2.67	97.36	0.7575	5.92
Proposed	0.68	0.88	1.56	98.38	0.8385	6.89
