# Erratum: Lou, X.; Jia, Z.; Yang, J.; Kasabov, N. Change Detection in SAR Images Based on the ROF Model Semi-Implicit Denoising Method. *Sensors* 2019, *19*, 1179

**DOI:** 10.3390/s19102314

**Published:** 2019-05-20

**Authors:** Xuemei Lou, Zhenhong Jia, Jie Yang, Nikola Kasabov

**Affiliations:** 1 College of Information Science and Engineering, Xinjiang University, Urumuqi 830046, China; lxm07240112@163.com; 2 Institute of Image Processing and Pattern Recognition, Shanghai Jiao Tong University, Shanghai 200240, China; Jieyang@sjtu.edu.cn; 3 Knowledge Engineering and Discovery Research Institute, Auckland University of Technology, Auckland 1020, New Zealand; nkasabov@aut.ac.nz

The authors wish to make the following erratum to this paper [1]:

The corresponding author should be Zhenhong Jia instead of Xuemei Lou. The corrected correspondence information is “jzhh@xju.edu.cn; Tel.: +86-0991-8583-362”.

The authors would like to apologize for any inconvenience caused to the readers by these changes.
